# Earth Mover’s Distance (EMD): A True Metric for Comparing Biomarker Expression Levels in Cell Populations

**DOI:** 10.1371/journal.pone.0151859

**Published:** 2016-03-23

**Authors:** Darya Y. Orlova, Noah Zimmerman, Stephen Meehan, Connor Meehan, Jeffrey Waters, Eliver E. B. Ghosn, Alexander Filatenkov, Gleb A. Kolyagin, Yael Gernez, Shanel Tsuda, Wayne Moore, Richard B. Moss, Leonore A. Herzenberg, Guenther Walther

**Affiliations:** 1 Department of Genetics, Stanford University School of Medicine, Stanford, California, United States of America; 2 Department of Statistics, Stanford University, Stanford, California, United States of America; 3 Department of Mathematics, California Institute of Technology, Pasadena, California, United States of America; 4 Division of Immunology and Rheumatology, Stanford University School of Medicine, Stanford, California, United States of America; 5 Independent Researcher, Menlo Park, California, United States of America; 6 Department of Allergy and Immunology, Mount Sinai Hospital, New York, New York, United States of America; 7 Center for Excellence in Pulmonary Biology, Stanford University School of Medicine, Stanford, California, United States of America; Virginia Tech University, UNITED STATES

## Abstract

Changes in the frequencies of cell subsets that (co)express characteristic biomarkers, or levels of the biomarkers on the subsets, are widely used as indices of drug response, disease prognosis, stem cell reconstitution, etc. However, although the currently available computational “gating” tools accurately reveal subset frequencies and marker expression levels, they fail to enable statistically reliable judgements as to whether these frequencies and expression levels differ significantly between/among subject groups. Here we introduce flow cytometry data analysis pipeline which includes the Earth Mover’s Distance (EMD) metric as solution to this problem. Well known as an informative quantitative measure of differences between distributions, we present three exemplary studies showing that EMD 1) reveals clinically-relevant shifts in two markers on blood basophils responding to an offending allergen; 2) shows that ablative tumor radiation induces significant changes in the murine colon cancer tumor microenvironment; and, 3) ranks immunological differences in mouse peritoneal cavity cells harvested from three genetically distinct mouse strains.

## Introduction

The ability to measure differences in antigen expression on subsets of cells contributes to our understanding of clinical outcomes such as drug response, disease susceptibility, and prognosis [1]. Simultaneous flow measurements of dozens of characteristics on millions of cells in a sample are now routinely possible. However, methods for quantitatively comparing the multivariate non-parametric flow cytometry data distributions generated in clinical and biomedical settings lag sorely behind. This considerably compromises the utility of high dimensional (Hi-D) flow measurements for clinical and research purposes.

Flow cytometry is a good example as well as a key one needing current attention. At present, flow cytometry methods for detecting and monitoring Hi-D differences between/among samples are largely based on determination of whether the median levels of markers shift and/or whether cells “move” from one gate to another. However, as Bernas et al. point out [2]: “an accurate comparison of any pair of histograms [populations] should involve an application of reproducible measure of (dis)similarity and an estimation of the statistical significance of such a measure”.

Extending this (Bernas et al. [2]) to be informative for biological and medical studies, the (dis)similarity measure should satisfy the following criteria: (1) it must possess the properties of a metric (see below); (2) it should distinguish biologically significant differences from small differences due to instrument drift or other irrelevant factors; (3) it should be non-parametric to account for the complex structure of the cell populations commonly found in flow cytometry data; and, (4) it should be computationally efficient so that modern high throughput analyses can be performed quickly.

While the comparison of two populations (histograms) is a well-studied theme with a plethora of methodologies available in the literature [3–9], requirement #2 (above) basically rules out these approaches for flow cytometry and similar datasets, where very minimal shifts in flow instrument configuration during data collection commonly cause minor data aberrations that can appear to be statistically significant (albeit not biologically important) differences between samples. That is, if the sample size is large enough, approaches based on statistical significance (p-values) will typically report even small shifts as highly significant, once again recalling the well-known slogan ‘*statistically significant* does not necessarily mean *practically meaningful*’ e.g., see [2, 10–14].

As argued by Bernas et al. [2] and Zimmerman [14], attention should focus on appropriate distance metrics, defined as non-negative symmetric functions that satisfy the triangle inequality and the axiom of coincidence [15]. Not all distance metrics, however, satisfy requirement #2, i.e., are insensitive to small changes. For example, metrics that compare frequencies in bins typically do not satisfy this requirement, i.e., if the frequencies in adjacent bins are different and a small location change simply shifts the contents by one bin, then the observed discrepancy between the two shifted samples can be large. Except for Earth Mover’s Distance (EMD), all of the metrics listed in Bernas et al. [2] Table 2 fall into this category.

**Fig 1:**
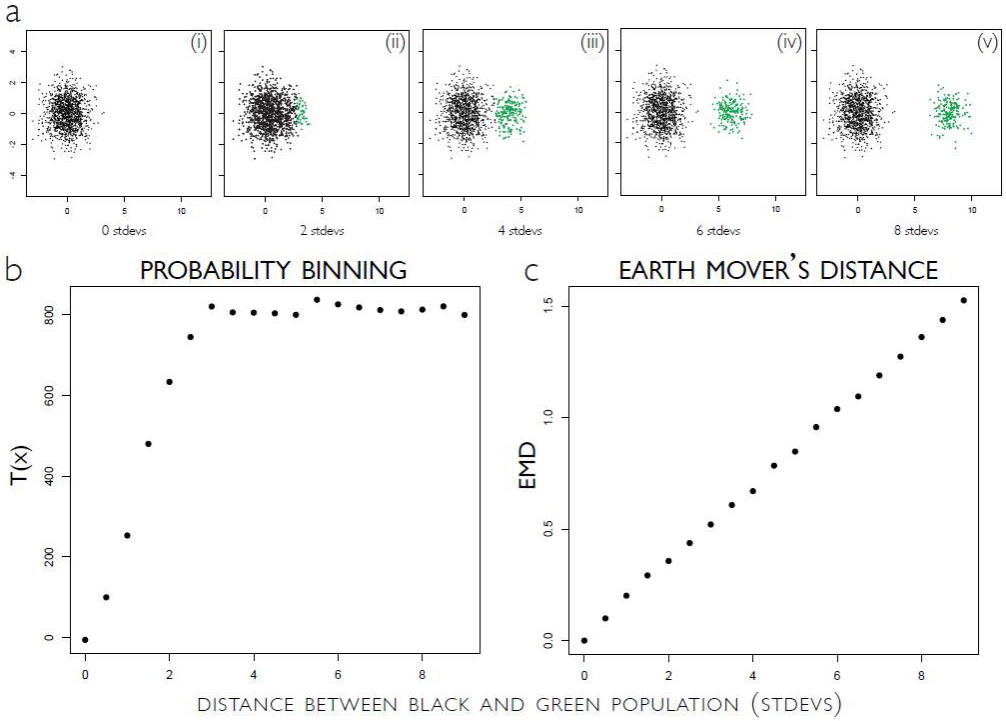
EMD score increases linearly with the growing separation between two populations. Panel (a) of Fig 1 shows two normal distributions: a large population (black) and a smaller population (green). The green population starts with a mean at the same position as the black population, and increases along the x axis in fixed increments (2 standard deviations) in each of the successive panels. At each step, we calculate the probability binning (PB) statistic (T (χ)) [7], which is based on p-values, and the Earth Mover’s Distance (EMD, described in detail Results section) between the “unstimulated” first panel in (a), and the joint distribution of the main (black) population with stimulated population (green). As the green population moves further from the black population, both the PB and the EMD increase monotonically. However, when the green population gets past 2 standard deviations from the black population, the PB plateaus as the two distributions have reached “maximum” separation based on the PB statistic. No additional movement of the green population will provide further evidence about the hypothesis that these two populations are the same, while a larger separation clearly carries biologically relevant information. Conversely, EMD continues to increase linearly with the growing separation of the green population. This example illustrates the two shortcomings of using p-values to quantitate change: Even a small change which may not be meaningful can be highly significant and thus produce a large value of the PB statistic, and larger changes may not increase this statistic further although from a biological point of view it would be desirable to do so. While the data for this figure were generated synthetically, one can imagine an experiment in which increasing amounts of a drug are applied causing a subset of cells to increase expression of a marker based on the amount of the drug. In order to correlate the amount of drug with the level of expression in a reliable fashion, one needs a true distance metric to measure the magnitude of the change in distributions. This figure appeared originally in the PhD thesis written by one of the authors (Noah Zimmerman), which was accepted in 2011 by Stanford University and is available online [14]. Reprinted from [14] under a CC BY license, with permission from Noah Zimmerman, original copyright 2011.

EMD satisfies this requirement (#2, above) for being insensitive to instrument drift and other small changes because, in an abstract sense, EMD is a product of changes in location *and* frequency rather than just changes in one or the other. Therefore, small changes in either subset location (e,g., due to instrument drift) or subset frequency will be reflected as small changes in the EMD score (see Fig 1). This property, together with recent advances in the computation of EMD [16], make it well suited for the analysis of Hi-D flow cytometry data.

EMD was originally invented to solve certain kinds of transporting problems [16]. However, as we show here, it can be fruitfully applied to quantifying biologically informative differences between/among flow cytometry samples. Thus, if one thinks of a flow cytometry cell distribution as a pile of dirt, then the EMD between two distributions is the minimum cost required to move the dirt in one pile to the other. Here, cost is defined as the amount of dirt moved multiplied by the distance it is moved. In this context, the biological interpretation of the EMD between two flow cytometry samples involves both the proportion of cells whose marker expression has changed, and the magnitude of the change. This is a key advantage of the EMD over other metrics such as the quadratic form metric established by Bernas et al. [2] or the bipartite matching introduced by Pyne et al [9].

Practically, EMD can be computed quickly with the ‘Hungarian algorithm’ [16]. Further, since EMD has been employed successfully in other areas such as computer vision [17], experience with various implementations of the code is readily available. Finally, the EMD analyses that we present here can be used with any method that enables identification and isolation of cellular subsets on/in which markers are expressed. For example, as we demonstrate here, EMD can be readily computed in combination with the density-based merging (DBM) clustering method that we have developed [18].

Here we demonstrate the practical utility of EMD (as part of the flow cytometry data analysis pipeline) through three proof-of-concept studies (see below). The first is an allergy study, in which EMD was used to determine clinically-relevant shifts in the expression of basophil surface markers (CD203c and CD63), whose expression is known to increase following hypersensitivity reaction to the *Aspergillus fumigatus* (*A*. *fumigatus*) allergen [19]. The second is an application of the EMD approach to quantifying changes in the murine colon cancer tumor immune cell microenvironment in response to ablative tumor radiation. The last application that we present is the use of EMD to quantify phenotypic differences between three commonly used, genetically distinct mouse strains.

## Results

### The EMD algorithm for flow cytometry data analysis

We compute the EMD for comparing two cell populations that are defined either by a manual or an automated gating algorithm. First we compute signatures [20], which are histogram-like approximations of the data, for each of the two cell population. There are published methods for generating such signatures for image data [20]. However, generating the signatures for flow cytometry data requires a different approach, which we describe below. Once the signatures are generated, the EMD computation can be stated in terms of a linear programming problem.

#### Signatures

For efficiency, distributions are summarized by signatures, which allow for more granularity in high-density areas of the data, and less granularity in sparse areas, i.e., signature bins are variable in size whereas histogram bins are typically derived from a fixed size partitioning of the distribution,

Formally, a signature {*s*_*j*_ *= (m*_*j*_, *w*_*mj*_*)*} is represented by the mean (*m*_*j*_) of a group *j* of observations, and the fraction (*w*_*mj*_) of observations that belong to the group *j*. To bin the data into the groups used in the signature, we use the binning algorithm described by Roederer *et al*. [7]. Thus, we first calculate the variance of the data for each of the parameters (dimensions) included in the analysis. Then, we chose the dimension with the largest variance, and split the events into two bins along the median value in that dimension such that half of the events fall in each of the two resulting bins. We then proceed recursively until a pre-defined threshold is met, e.g., *2ln(N)* observations per bin, where *N* is a total number of events. For each bin to be split, the algorithm chooses the dimension that maximizes variance, splitting the data about the median value in that dimension. The result is a series of n-dimensional hyper-rectangular bins, each containing an equal number of events.

#### Computing EMD

Formally, EMD can be stated in terms of a linear programming problem: two distributions represented by signatures, *P = {(p*_*1*_,*w*_*p1*_*)*,*…*,*(p*_*m*_,*w*_*pm*_*)}* and *Q = {(q*_*1*_,*w*_*q1*_*)*,*…*,*(q*_*n*_,*w*_*qn*_*)}* where *p*_*i*_,*q*_*i*_ are bin centroids with frequencies *w*_*pi*_,*w*_*qi*_, and *D = [d*_*ij*_*]* the matrix containing the Euclidean distances between *p*_*i*_ and *q*_*j*_ for all *i*,*j*. We ensure that *P* and *Q* have the same total mass of unity (equal to 1) by normalizing each of the two distributions. Next, we find a flow *F = [f*_*ij*_*]* between *p*_*i*_ and *q*_*j*_ that minimizes the total cost

$$Cost(P,Q,F)=\sum_{i=1}^{m}\sum_{j=1}^{n}d_{ij}f_{ij}$$ (1)

subject to the following constraints:

$$f_{ij}\geq 0\;1\leq i\leq m,\;1\leq j\leq n$$ (2)

$$\sum_{j=1}^{n}f_{ij}=w_{p_{i}}\;1\leq i\leq m$$ (3)

$$\sum_{i=1}^{m}f_{ij}=w_{q_{j}}\;1\leq j\leq n$$ (4)

$$\sum_{i=1}^{m}\sum_{j=1}^{n}f_{ij}=\sum_{i=1}^{m}w_{p_{i}}=\sum_{j=1}^{n}w_{q_{j}}=1$$ (5)

Constraint (2) ensures that mass is only transported in one direction (e.g., from the source sample to the destination sample). Constraints (3) and (4) limit the amount of mass that can be moved from/to a given signature bin to their respective weights; and, constraint (5) ensures that the amount of mass moved does not exceed the maximum possible amount.

In the case of signatures with the same total mass, EMD is a true metric for distributions and is equivalent to the Mallow’s distance [21] as demonstrated by Levina and Bickel [22]. Thus, when applied to probability distributions, EMD has a clear probabilistic interpretation as the Mallow’s distance (in the applications described here, we ensure equal mass of two samples but retain the EMD notation.).

Solving the above linear programming problem determines the optimal flow, *F*, between the source and destination signatures subject to constraints (2–5). Then EMD is defined as a function of the optimal flow *F = [f*_*ij*_*]* and the ground distance *D = [d*_*ij*_*]* [22]:

$$EMD(P,Q)=\frac{\sum_{i=1}^{m}\sum_{j=1}^{n}d_{ij}f_{ij}}{\sum_{i=1}^{m}\sum_{j=1}^{n}f_{ij}}$$ (6)

EMD performance for flow data analysis was initially examined by Zimmerman, who reports the robustness of EMD performance in terms of binning parameters and sample size in his doctoral thesis [14]. Comparisons of small populations of cells with low frequencies may require finer binning than comparisons of larger populations. However, as Zimmerman [14] shows, overall EMD is robust regardless of the choice of the number of bins.

### EMD comparisons are useful in a wide range of biomedical studies

**Fig 2:**
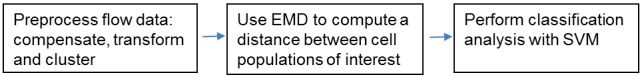
Data analysis workflow for application of EMD to flow cytometry data. Flow data were collected with H-D flow instruments available in the Stanford Shared FACS Facility and preprocessed with AutoGate software (freely available at http://CytoGenie.org). The third step (classification analysis was applied only to the first two studies described in the Results section).

The same data analysis workflow (Fig 2) was used in the three examples discussed below.

#### EMD, a diagnostic tool to distinguish CF patients from patients with CF-ABPA

**Fig 3:**
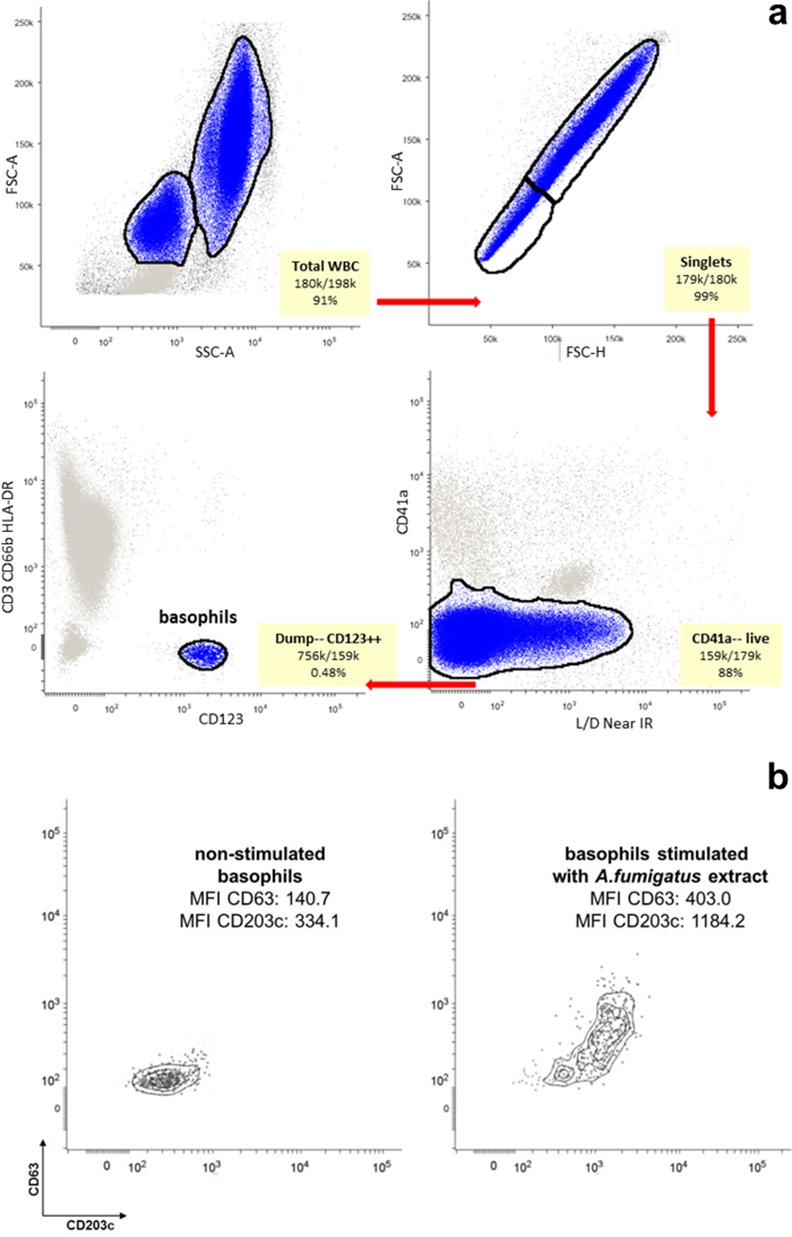
Analysis of basophils activation status. **(a)** To identify basophils, we used the following gating sequence (shown in the figure by the red arrows): FSC-A/SSC-A (total white blood cells)→ FSС-A/FSC-H (singlets)→ CD41a/live/dead (CD41a—live)→ Dump [CD3, CD66b, HLA-DR]/CD123 (Dump—, CD123++)→ use EMD with CD203c/ CD63 [19] to determine basophil activation status. **(b)** An example of basophils response to stimulation with the *A*. *fumigatus* allergen. Here, MFI represents median fluorescence intensity.

In this study [19, 23], the authors co-measure surface levels of CD203c and CD63 on peripheral blood basophils following *in vitro* stimulation with *A*. *fumigatus* allergen/extract (see Fig 3B) and test whether increases Median Fluorescence Intensity (MFI) for each marker distinguish CF-ABPA from CF patients (clinical status was confirmed by conventional clinical diagnostic techniques). They report that CD203c increases distinguish CF patients allergic to *A*. *fumigatus* (CF-ABPA) from those who are not allergic to the fungus. They also showed modest upregulation of CD63 in the allergic patients, but did not find a statistically significant difference for the expression level of this marker between the two patient groups.

**Fig 4:**
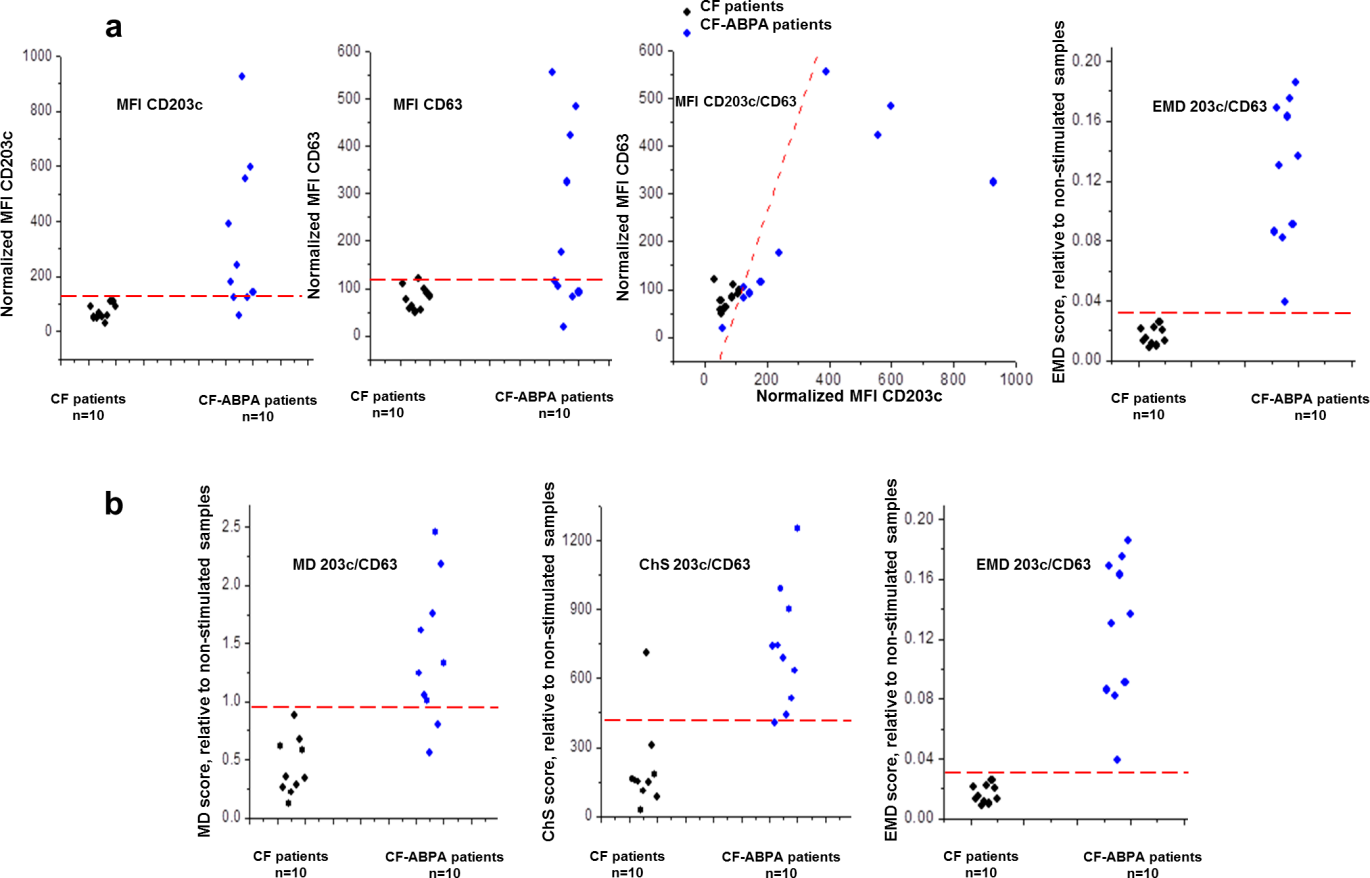
EMD scores based on expression of two independent flow cytometry markers more accurately distinguish allergic (CF-ABPA) from non-allergic (CF) patients. **(a)** This panel compares EMD scores for the combined expression of CD203c and CD63 with “classical” median fluorescence intensity (MFI) values computed separately for the expression of each marker and with MFI values computed for the combined expression of CD203c and CD63. **(b)** Performance comparison between EMD and two other representative “metrics”, one based on test statistics, Chi-Square (ChS) [2] and the other is a distance measure, Mahalanobis Distance (MD). All six measures were calculated relative to each sample’s unstimulated control. Data are shown for 20/45 CF patients (10 with CF-ABPA and 10 with only CF) drawn from a previously published CF-ABPA study [19, 23] and selected as described in Materials and Methods. For each CF patient sample (n = 10) and CF-ABPA (n = 10), we calculated the EMD on the CD63 and CD203c channels between the unstimulated controls and samples stimulated with the *A*.*fumigatus* allergen/extract. Using the SVM method we then defined thresholds (red dashed lines at 123.67 for MFI CD203c, 112.75 for MFI CD63, 0.92 for MD CD203c/CD63, 426.66 for CD203c/CD63 ChS and 0.03 for EMD CD203c/CD63) to distinguish allergic/positive responses from non-allergic/negative responses, i.e., we tried all possible combinations of 5 CF and 5 CF-ABPA patients and used the scores in each case as a training set to find an SVM threshold that divides the dataset in two categories (CF and CF-ABPA) with the lowest possible misclassification rate.

Here, we show EMD scores for the combined expression of CD203c/CD63 provide a more rigorous basis for patient classification than MFI values based either on CD203c or CD63 measurements, or MFI values based on CD203c/CD63 (Fig 4A). We also demonstrate EMD superiority over two representative “metrics” (Fig 4B), one based on test statistics, Chi-square (ChS) [2] and the other a well-known distance measure, Mahalanobis Distance (MD) [8]. For the EMD measure shown in the figure, we computed the *EMD(c*,*p)* score for each sample using Euclidean distance as the ground distance *D* (see “Computing EMD”), where *c* is the unstimulated control, *p* is the sample stimulated with the offending allergen (see Fig 4). Then, for all six measures, we applied the support vector machine method (SVM) [24] to the data for half the patients to define thresholds that distinguish the CF from CF-ABPA groups. We then applied these thresholds to classify the remaining patients (Fig 4).

To validate the SVM threshold values and estimate their accuracy, we used a method equivalent to repeated random sub-sampling that randomly splits each dataset (CF and CF-ABPA) into two datasets: training and validation. Since the possible disadvantage of this method is that some observations may never be selected in the validation subsample, we artificially assured that all observations will be selected at least once in the validation subsample. Such repeated random sub-sampling revealed a training error for the best classification of the validation dataset in each analysis type: 1/10 patients for MFI CD203c; 5/10 patients for MFI CD63; 1/10 patients for MFI CD203c/CD63; 2/10 patients for MD CD203c/CD63; 2/10 patients for ChS CD203c/CD63 but only 0/10 patients for EMD CD203c/CD63. These findings clearly demonstrate that EMD offers a superior robust measure of responsiveness that is not bound to changes in expression levels of a single marker.

#### Monitoring tumor response during radiotherapy: mice with colon cancer

**Fig 5:**
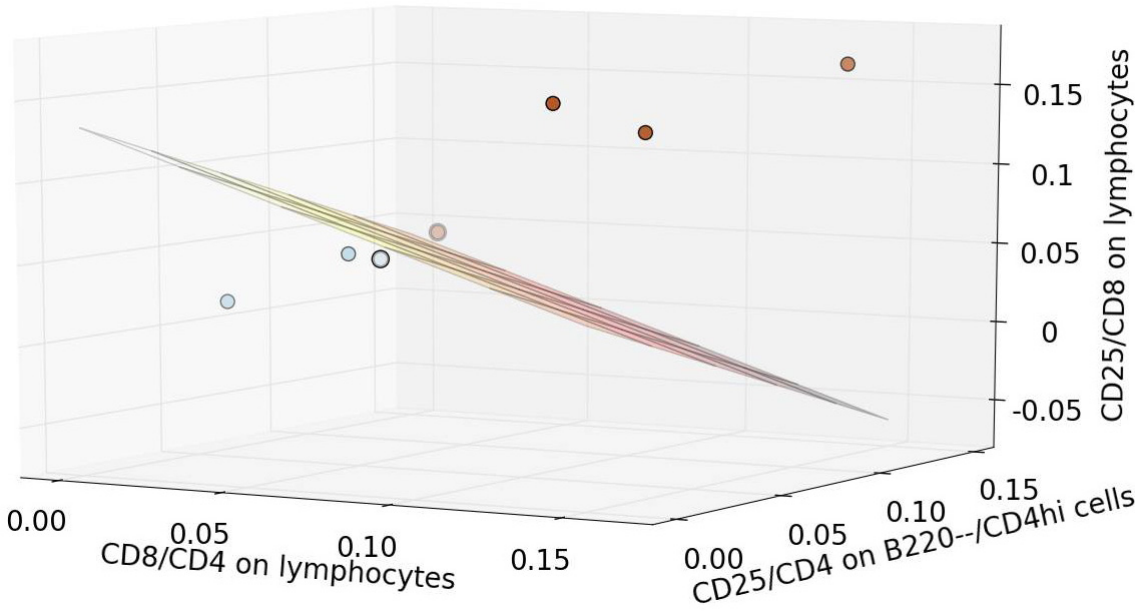
Combined EMD score for three pairs of biomarkers distinguishes mice that received tumor radiotherapy from untreated tumor-bearing mice. Tumor infiltrating cells from tumor-bearing mice that received tumor radiotherapy (n = 4, red dots) or were untreated (n = 3, blue dots) were gated according to three different strategies: CD4/CD8 expression on live lymphocytes (dead—/ SSC-A; FSC-A/SSC-A); CD25/CD4 expression on B220—/CD4hi live lymphocytes; and, CD25/CD8 expression on live lymphocytes. We then calculated three EMD scores for the following combinations of biomarkers and cell populations: CD8/CD4 expression on lymphocytes, CD25/CD4 expression on B220—/CD4hi lymphocytes, CD25/CD8 expression on lymphocytes. The EMD scores were calculated relative to the control sample (mouse which did not receive tumor radiotherapy). The threshold (plane) was defined using the SVM method.

We applied EMD to data acquired in a study evaluating the role of tumor immunity in a mouse model in which high-dose, single fraction tumor radiation induces complete durable remission [25]. Since the number and surface marker expression of cells in the tumor microenvironment were measured in this study, we used EMD to quantify changes in the balance between critical immune cell types (CD4, CD8, CD25 cells, etc.) in response to the radiotherapy (see Fig 5). This approach, which allows biologically meaningful quantitative comparisons of multivariate flow cytometry data can potentially be used for quantitative systematic examination of the tumor immune microenvironment.

#### Quantifying immunological and phenotypic differences among three genetically distinct mouse strains

**Fig 6:**
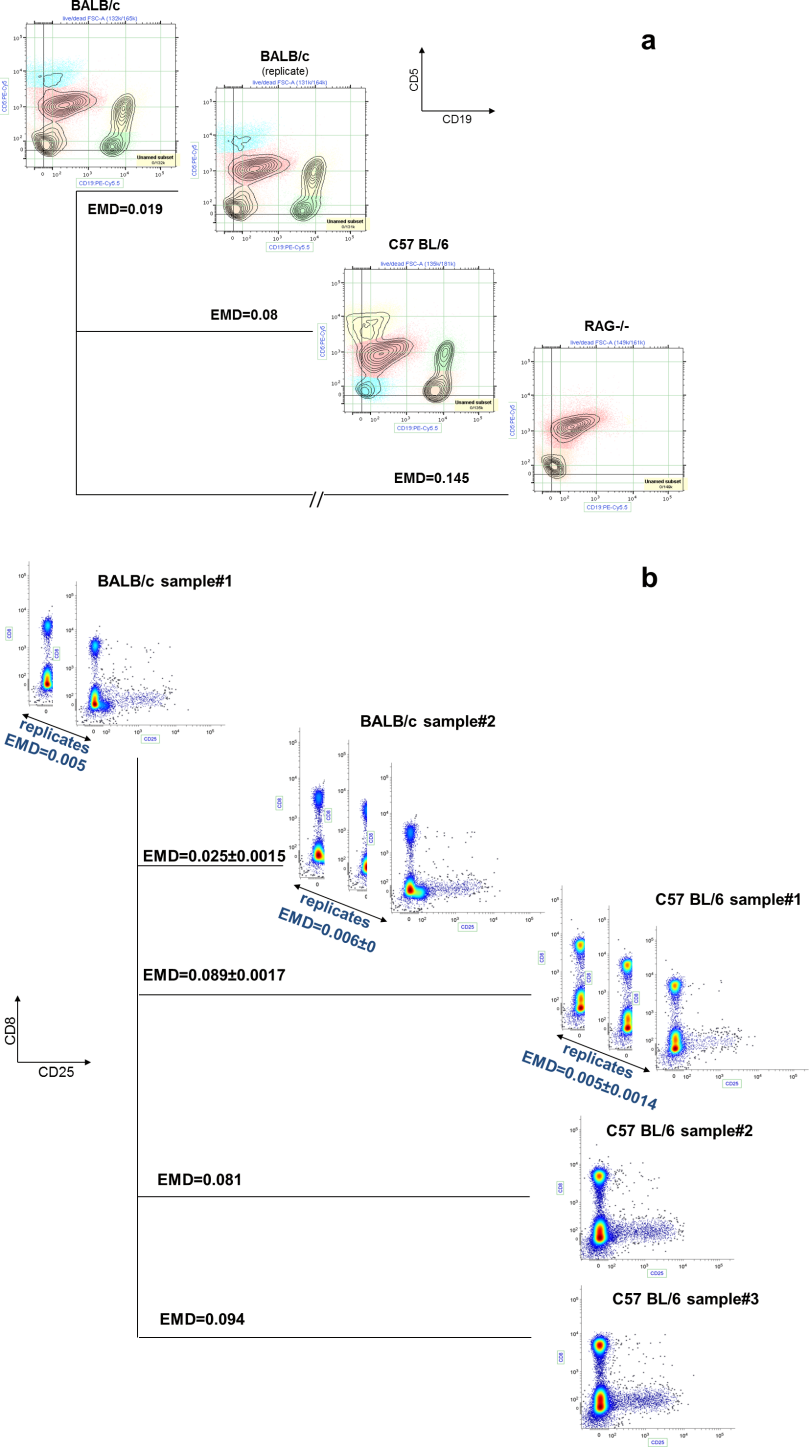
EMD scores detect relatively small differences between wild-type strains and detect the much larger differences between the wild-type mice and knockout mice. **(a)** We used the following gating strategy (according to [26]): dead—/FSC-A→FSC-H/FSC-A. Then we performed the EMD comparison for the expression level of CD19/CD5 biomarkers on peritoneal cells among three mouse strains. EMD values represent the EMD scores between the first BALB/c sample (reference) and the other 3 subsequent samples, including a replicate for BALB/c cells. **(b)** Mouse spleen cells from BALB/c and C57BL/6 mice stained and analyzed using 13-parameter high-dimensional FACS. We then used the following gating strategy: FSC-H/FSC-A (singlets)→dead—/SSC-A (live). Each plot displays the expression level of CD8 and CD25 for spleen cells from corresponding mouse strain. On this figure, replicate is the same sample which was run several times. EMD scores for inter-sample variability are significantly lower than EMD scores for inter-strain variability.

We used EMD to rank differences among peritoneal cavity cells from three mouse strains based on their similarities to a reference sample obtained from a mouse in one of the three strains (BALB/c). Using the flow cytometry measurements for the CD5 and CD19 surface markers (which respectively identify T and B lymphocytes), we computed EMD scores comparing the data for the reference sample and for samples obtained from each of the three mouse strains (Fig 6). Based upon the known genetic backgrounds of the mice, we predicted that the “correct” ordering of the strains is BALB/c→BALB/c replicate→С57BL/6→RAG -/-. The EMD scores that we obtained were consistent with this order.

As expected, the EMD score computed between the reference sample and a second sample from the same animal (BALB/c replicate) was the smallest. The next nearest EMD score was computed between genetically distinct wild type animals (C57BL/6 and BALB/c). The highest EMD score was computed between BALB/c and a BALB/c genetic “knock-out” strain (RAG- /-) that lacks all lymphocytes. These results corroborate the existing understanding of “similarity” in genetic backgrounds among these three mouse strains [26–28].

### Comparison between EMD, Probability Binning (PB), Chi-Square (ChS) and Mahalanobis Distance (MD)

**Fig 7:**
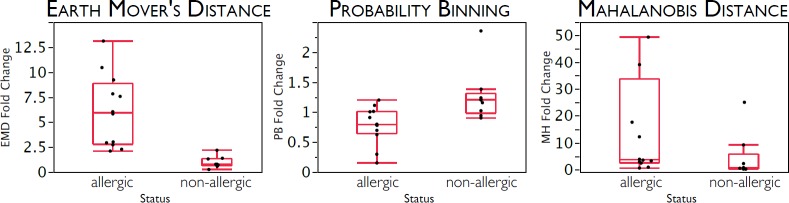
EMD clearly differentiates allergic (n = 13) and non-allergic (n = 9) groups of samples, while PB and MD are not able to differentiate them with sensitivity and specificity comparable to EMD. This figure appeared originally in the PhD thesis written by one of the authors (Noah Zimmerman), which was accepted in 2011 by Stanford University and is available online [14]. The data come from a peanut allergy study by Gernez et al [31].Fold change calculated as a ratio of EMD (PB or MD) between unstimulated sample and sample stimulated with offending allergen, normalized by the EMD (PB or MD) between unstimulated sample and sample stimulated with non-offending allergen. Reprinted from [14] under a CC BY license, with permission from Noah Zimmerman, original copyright 2011.

While EMD has theoretical advantages over other methods proposed for population comparison (see Introduction), the empirical comparison in Figs 4B and 7 demonstrate EMD superiority over three representative “metrics”, two based on test statistics, Chi-Square (ChS) [2] and Probability Binning (PB) [7], and the other is a commonly used distance measure, Mahalanobis Distance (MD) [8]. Comparing EMD results with results obtained when these metrics are applied to the same data set shows that while EMD clearly distinguishes the allergic from non-allergic groups of samples, neither ChS and PB nor MD are able to distinguish these subject groups with the comparable sensitivity and specificity. However, more robust comparison would require benchmark datasets of larger size.

### EMD scope

EMD works well with flow cytometry data and similar types of datasets reporting data for continuous variable that are either parametrically or non-parametrically distributed. It can readily be applied to comparisons of pairs of distributions that are the same size or widely disparate in size. In addition, as Zimmerman shows [14], the overall EMD method is robust with respect to the choice of number of bins, although rare and/or sparsely-distributed populations may require finer binning.

As configured in the software used here, EMD readily compares responses measured in one or two dimensions. Extension to additional dimensions is theoretically possible. However, application of parallel programming algorithms and other optimization method will likely be required in this case.

## Discussion

Most studies that use flow cytometry measurements focus on identifying and/or quantitating biologically meaningful differences. For example, in the allergy example described here, we asked “Do samples from patients with CF-ABPA differ in a meaningful way from samples from CF patients?” and “How relatively large is the difference between samples from the two groups of patients?” Studies here demonstrate that, as suggested by Bernas et al [2] and Zimmerman [14], questions such as these should be framed in terms of the *distance between samples* rather than the statistical significance (p-values) of the difference between the samples.

Our studies introduce Earth Mover’s Distance (EMD) as a measure that is well suited to quantitating multivariate changes in biomarkers measured, in this case, by flow cytometry. We demonstrate the practical utility of this EMD measure (as part of the flow cytometry data analysis pipeline) in three studies:

1. An allergy study, in which we show that EMD analysis of poorly coordinated shifts in the expression of two independent flow cytometry-measured basophil surface markers (CD203c and CD63) readily distinguishes patients allergic to a fungus from those who are not.
2. A comparative study of lymphocyte subset representation in three well-studied mouse strains (BALB/c, C57BL/6 and RAG-/-), where we show that EMD scores are sensitive enough to detect relatively small differences in lymphocyte frequencies between the two wild-type strains, BALB/c and C57BL/6, and readily detect much larger differences between wild-type mice and RAG -/- “knockout” mice, which completely lack lymphocytes.
3. A study of the efficacy of radiotherapy regimen in a mouse model of colon cancer, where we show that calculating the EMD scores before and after ablative tumor radiation provides a measure of the success of the ablative therapy.

Together, these findings indicate that the EMD approach can be used to sensitively detect differences in the degree and quality of an immune response, for example in lymphoid cells harvested before and after vaccination therapy, or in other cell types responding to allergens or treatments intended to decrease allergic responses. Thus, by extrapolation, studies reported here suggest that EMD analyses can improve outcomes obtained with any flow cytometry, mass cytometry, or other technology that reports distributions of measurements.

We have already made a version of the EMD approach available in the AutoGate software developed by our laboratory (available free of charge to.edu,.gov and.org users, see CytoGenie.org) to enable clinicians and researchers to apply it to past or future flow cytometry data and, in the future, to other situations in which accurately recognizing quantitative differences between samples is key.

## Materials and Methods

### Experiment overview

We use EMD to quantify differences between relevant subsets within the three biological/biomedical datasets described below.

### Flow samples and specimens description

All of the three biological/biomedical flow cytometry datasets utilized here were generated in previously published studies (see [19, 23, 25, 26] for complete details). Briefly, the data sets were stored immediately after collection into a stable long-term archive maintained by the Stanford Shared FACS Facility. Access to the data was provided by the investigators responsible for the studies. Human subject guidelines were followed in Gernez et al. [19, 23]; the other two studies used mouse data collected under IRB review. Patient records/information was anonymized and de-identified prior to analysis. The Stanford University Committee on Animal Welfare (APLAC) approved mouse protocol used in this study (protocol # 26768; protocol director: Strober; department: Medicine—Med/Immunology & Rheumatology; protocol title: Radiation and immunotherapy therapy for solid tumors).

Cell preparation and staining: For all of the samples analyzed, single cell suspensions were prepared, stained with fluorochrome-coupled reagents, and analyzed with a high-dimensional flow cytometry instrument in the Stanford Shared FACS Facility. Cell preparation and staining methods varied according to the needs of the individual studies and are described in detail in study [19, 23, 25, 26]. Please see figure legends for gating sequences and other details specific to each analysis.

#### The human CF-ABPA

These studies were designed to distinguish between cystic fibrosis (CF) patients who either did, or did not, have allergic bronchopulmonary aspergillosis (ABPA), a severe complication in CF. Complete materials and methods for this study can be found in [19, 23]. Work reported here is based on flow cytometry data for a total of de-identified 20 patients, 10 of whom were randomly selected from clinically diagnosed as CF-ABPA group (20 patients total in the original study); the other 10 were randomly selected from those diagnosed as ABPA-free (13 CF patients colonized with *A*. *fumigatus* and 12 CF patients free of the fungus in the original study). Of these, 3 were shown by the original study to be colonized with *A*. *fumigatus* while 7 were shown to be free of the fungus.

The dataset for these 20 patients is available at https://flowrepository.org/id/FR-FCM-ZZJQ

#### The murine colon cancer study

This study was designed to elucidate the role of tumor immunity in a mouse model in which high-dose, single fraction tumor radiation induces complete durable remissions [25]. Materials and methods for this study can be found in [25]. Briefly, we compared flow cytometry data sets collected individually for eight mice carrying a CT26 colon tumor (>1 cm in diameter). Four of the mice were irradiated and four served as non-irradiated controls. The dataset used here is available at **https://flowrepository.org/id/FR-FCM-ZZJE**

#### The mouse peritoneal cavity macrophage study

This study was designed to contrast the phenotypes of cells from three genetically distinct mouse strain, two of which are commonly used wild type strains (BALB/c and C57BL/6), while the third is a genetic knockout strain (BALB/c RAG-/-) that fails to develop lymphocytes but show normal myeloid cells and granulocyte development. Complete materials and methods for this study can be found in [25]. The data sets used in this manuscript are available at https://flowrepository.org/id/FR-FCM-ZZJF and https://flowrepository.org/id/FR-FCM-ZZKE

### Instrument details

Information about instruments used to collect the datasets used here can be found in [19, 23, 25, 26].

### Data analysis details

The proposed workflow for analyzing all three datasets used in this manuscript consists of three steps (see Fig 2):

1. Preprocess the data by sequentially using utilities available in AutoGate [29] (http://CytoGenie.org/) to generate compensated data, transform it with the Logicle transformation [30], and cluster the transformed data with DBM [18]. Please see figure legends for gating sequences. The flow cytometry data prepocessing methods used here do not require user input for parameter such as number of clusters, number of grid bins, density threshold, manual gating for compensation purposes, etc.
2. Use EMD to measure the changes in cell surface marker expression for cells of interest, for example, in defined populations of cells analyzed before and after stimulation or before and after treatment (depending on the biological/biomedical study, see example on Fig 3). Computing the EMD is a standard problem in the case of signatures (i.e., binned data). Here, we computed EMD with code available at http://www.mathworks.com/matlabcentral/fileexchange/22962-the-earth-mover-s-distance, which is integrated into AutoGate (http://CytoGenie.org/).
3. Finally, perform classification analysis using a support vector machine (SVM, linear kernel, no normalization was done) method implemented in Python scikit-learn 0.16.1 library. Given a set of training examples mapped as points in space, each marked as belonging to one of two categories, the SVM finds a maximal “gap” between the two categories and assigns new examples into one category or the other based on which side of the gap they fall on. To accommodate both non-linearly separated (MFI CD203c, MFI CD63, MFI CD203c/CD63, MD CD203c/CD63 and ChS CD203c/CD63) and linearly separated (EMD CD203c/CD63) data sets we ensured both soft (regularization parameter C = 1) and hard (C = 10^5^) margins respectively.

Combining Logicle transformation, DBM for cell population identification, probability binning, EMD, and SVM provides a complete pipeline for computational classification of flow cytometry samples. However we would like to emphasize that the EMD approach for quantitative comparison of flow cytometric histograms works independently of how the population was defined, e.g. by using domain knowledge-driven manual gating, sequential automated clustering approach or simultaneous clustering approach. When we compare EMD to other metrics, we use exactly the same pipeline for each metric.
